# Diabetic kidney disease: m6A modification as a marker of disease progression and subtype classification

**DOI:** 10.3389/fmed.2025.1494162

**Published:** 2025-03-04

**Authors:** Wenzhe Li, Gaosi Xu, Manna Li

**Affiliations:** ^1^The Second Clinical Medical College of Nanchang University, Nanchang, China; ^2^Department of Nephrology, Second Affiliated Hospital of Nanchang University, Nanchang, China

**Keywords:** diabetic kidney disease, epigenetics, N6-methyladenosine modification, immune characteristics, bioinformatic analysis

## Abstract

This paper aims to investigate m6A modification during DKD progression. We evaluated m6A regulators expression in peripheral blood mononuclear cells, whole kidney tissue, glomerular, and tubulointerstitial samples. CIBERSORT and single-sample gene set enrichment analysis analyzed glomerular immune characteristics. Logistic-LASSO regression were used to develop the m6A regulators model that can identify early DKD. Consensus clustering algorithms were used to classify DKD in glomerular samples into m6A modified subtypes based on the expression of m6A regulators. Gene set variation analysis algorithm was used to evaluate the functional pathway enrichment of m6A modified subtypes. Weighted gene co-expression network analysis and protein–protein interaction networks identified m6A modified subtype marker genes. The Nephroseq V5 tool was used to evaluate the correlation between m6A modified subtypes marker genes and renal function. DKD patients’ m6A regulators expression differed from the control group in various tissue types. DKD stages have various immune characteristics. The m6A regulators model with YTHDC1, METTL3, and ALKBH5 better identified early DKD. DKD was divided into two subtypes based on the expression of 26 m6A regulators. Subtype 1 was enriched in myogenesis, collagen components, and cytokine receptor interaction, while subtype 2 was enriched in protein secretion, proliferation, apoptosis, and various signaling pathways (e.g., TGFβ signaling pathway, PI3K/AKT/mTOR pathway, and etc.). Finally, AXIN1 and GOLGA4 were identified as possible biomarkers associated with glomerular filtration rate. From the viewpoint of m6A modification, the immune characteristics and molecular mechanisms of DKD at various stages are different, and targeted treatment would improve efficacy.

## Introduction

With the increase of population aging, poor living habits and environmental pollution, the prevalence of diabetes mellitus is rapidly increasing. The number of people with diabetes is 463 million in 2019, and it is expected that about 700 million people worldwide will have diabetes by 2045 (1, 2). Diabetic patients with chronic complications are common, among which kidney involvement is more common, and diabetic kidney disease (DKD) has become a major cause of end-stage renal disease (ESRD) (3). Renal damage caused by diabetes can involve almost all structures of the kidney, and once renal impairment occurs, it progresses faster than in patients with non-diabetic kidney disease. It has also been clinically observed that when DKD progresses to end-stage renal failure, patients have a worse long-term prognosis than patients with other kidney diseases, whether given dialysis or kidney transplantation. The health burden associated with DKD remains a major challenge for individuals, families and society. Therefore, early diagnosis and treatment to delay the occurrence and development of DKD are of great significance to increase the survival rate and improve the quality of life of DKD patients.

Urinary microalbumin is a common early monitoring indicator for patients with DKD, but about 28% of patients do not develop proteinuria during the progression of the disease, and this variation of the disease brings difficulties in early diagnosis (4–6). Currently, the main pathogenic mechanisms of DKD include hemodynamic changes, metabolic disorders and inflammatory responses, while inflammation and immune responses play a central role in disease progression (7, 8). The control of metabolic abnormalities such as glucose, blood pressure, and lipids alone is not enough to meet the treatment needs, and many DKD patients still progress to ESRD. Therefore, there is an urgent need to further understand the pathogenesis of DKD, to find better early diagnostic indexes, and to develop new biomarkers and potential targets at the molecular level for the prevention and treatment of DKD.

Although individual genetic susceptibility and familial aggregation are associated with the development of DKD, there is growing evidence that epigenetics, which regulates gene expression independently of genomic sequence, also plays an important role in the development of the disease (9, 10). Post-transcriptional modifications are receiving increasing attention in most fields of epigenetics. Among them, the most abundant and prevalent modification in eukaryotic mRNAs is the m6A modification, which is defined as methylation at the sixth N position of adenylate (11, 12). The m6A modification is dynamically reversible, and the level of modification is regulated by methyltransferases (“Writers”), demethylases (“Erasers”) and methylated reading proteins (“Readers”). m6A regulators are involved in a variety of biological functions, including tissue development, cell differentiation, circadian rhythms, and tumor progression (13). In addition, m6A modification has been confirmed to be involved in inflammation and apoptosis in DKD, which plays an important role in disease progression (14, 15). METTL14 is associated with the progression of DKD, according to a previous study by our group (16). Nevertheless, the research on post-transcriptional epigenetic modifications in DKD is still in its infancy. To further understand the mechanism of m6A modifications in DKD, this study attempted to elucidate the mechanism of DKD progression and identify therapeutic targets from the perspective of m6A modifications through bioinformatics analysis.

## Materials and methods

### Data selection and preprocessing

**Figure 1:**
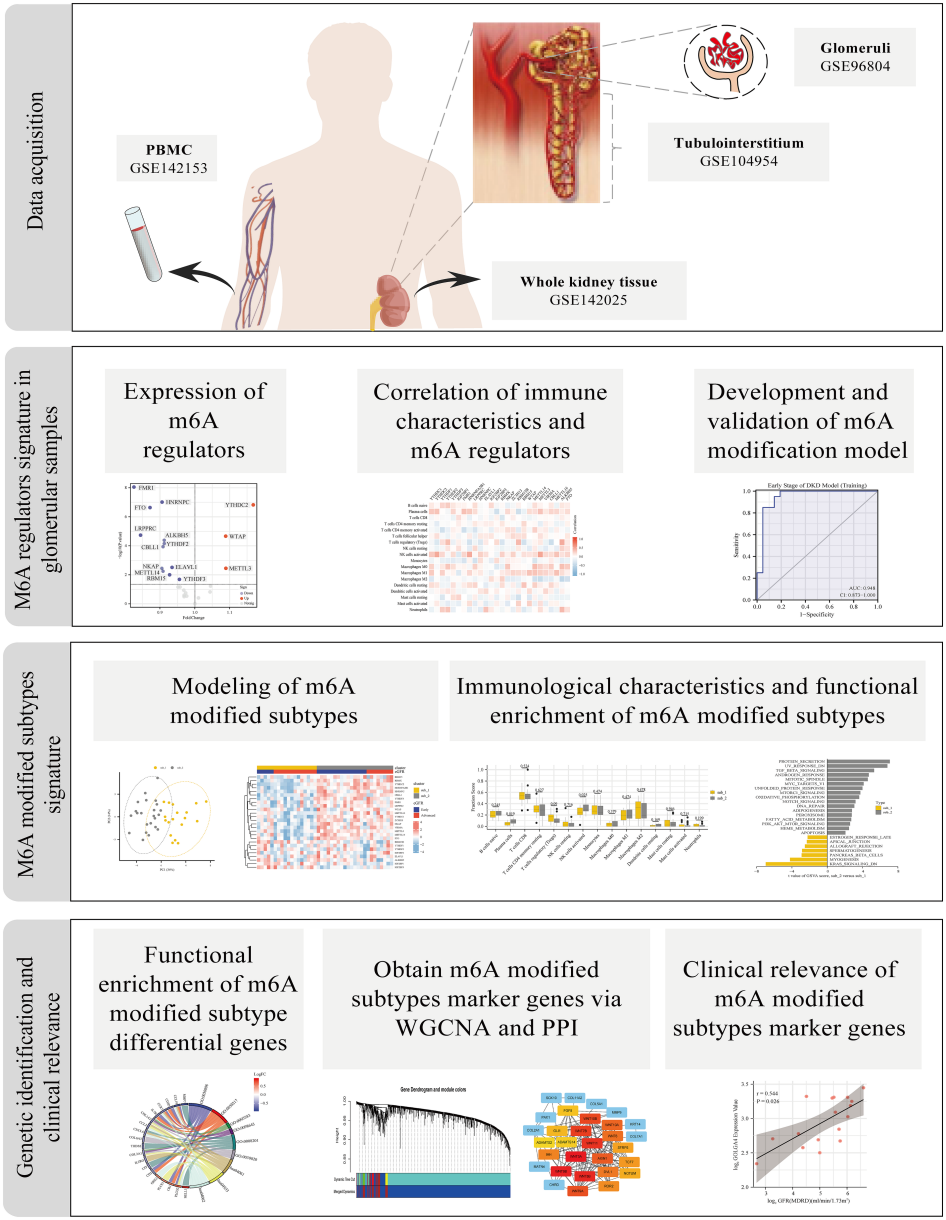
Study flow diagram. m6A, N6-methyladenosine; PBMC, Peripheral blood mononuclear cells; WGCNA, weighted gene co-expression network analysis; PPI, protein–protein interaction.

The flow chart of the study is shown in Figure 1. Relevant data were collected from the Gene Expression Omnibus (GEO) database^1^ for patients with DKD and controls. A total of four datasets were selected for this study: (I) GSE142153 is peripheral blood mononuclear cells (PBMC) sample data from GPL6480 (Agilent-014850 Whole Human Genome Microarray); (II) GSE142025 is the whole kidney tissue sample data from GPL20301 (Illumina HiSeq 4,000); (III) GSE96804 is microdissected glomerular sample data from GPL17586 (Affymetrix Human Transcriptome Array 2.0); (IV) GSE104954 is renal tubulointerstitial tissue sample data from GPL22945 (Affymetrix Human Genome U133 Plus 2.0 Array) and GPL24120 (Affymetrix Human Genome U133A Array).

Convert all probes to gene names, removing probes with no matching gene names or matching multiple gene names. When multiple probes existed for the same gene, the probe values were averaged. Raw data were normalized using the robust multi-array average (RMA) algorithm. Batch effects were removed with the combat function of the “SVA” R package (17).

### Evaluation of M6A regulators

The 27 m6A-modified regulators in this study were based on the results previously reported in the literature (18–21). The m6A regulators interaction network was analyzed using the interaction data from the STRING database^2^ and visualized using Cytoscape^3^ (22). The “limma” package was used to compare the differences in expression of m6A regulators between controls and DKD at different periods in different samples (23). Spearman correlation analysis was used to assess the expression relationship between m6A regulators in glomerular samples.

### M6A regulators and immune characteristics

Estimation of 22 types of infiltrated immune cells using the 1,000 permutation-based CIBERSORT algorithm in R (24). The immune-related gene cohorts were obtained from ImmPort database^4^. The immune response activity was evaluated by single-sample gene-set enrichment analysis (ssGSEA) algorithm in the “GSVA (gene set variation analysis)” R package. The level of immune cells infiltration and immune response activity between groups was assessed using differential expression heat maps or box plots, with *p* < 0.05 being a significant result. Spearman correlation analysis was used to assess the correlation between m6A regulators and immune characteristics in glomerular samples.

### Development and validation of M6A regulators model

In univariate logistic regression analysis, candidate m6A regulators with *p* < 0.05 were selected and included in the LASSO regression model. LASSO regression analysis was performed using the “glmnet” R package. The lambda value corresponding to minimized the cross-validated mean squared error was used for model selection (25). The m6A regulators with non-zero regression coefficients were selected by LASSO and further included in the multivariate logistic regression analysis. The final m6A regulators with *p* < 0.05 in the multivariate logistic regression model were used as diagnostic model classifier. We applied the classifier to the training and validation sets and evaluated the classifier performance based on area under curve (AUC) scores in the receiver operating characteristic curve (ROC).

### Unsupervised consensus clustering analysis of glomerular DKD samples

Based on the 26 identified m6A regulators in the glomerular DKD samples, a consensus clustering analysis was performed using the k-means algorithm in the “ConsensusClusterPlus” R package to identify the m6A modified subtypes. The consensus clustering algorithm was run for 1,000 iterations, with each iteration containing 80% of the samples to ensure the stability of the clustering. The optimal number of clusters is determined by the cumulative distribution function (CDF) curves of the consensus score and the consensus matrix heatmaps. The robustness of the k-values of the clustering analysis was verified by the “PCA (principal component analysis)” R package. The Kruskal-Wallis test was used to compare the expression of m6A regulators between subtypes.

### Functional pathway enrichment analysis of M6A modified subtypes

The expression matrix was transformed into a pathway activation score matrix using the “GSVA” R package, and the “limma” R package was used to compare the pathway activation scores between the two subtypes, with a *p* value <0.01 as the cut-off criterion (26). The gene sets “h.all.v7.5.1.symbols” and “c2.cp.kegg.v7.5.1.symbols” were downloaded from the MSigDB database^5^ and used for the GSVA analysis.

### Identification of genes and clinical significance

Differentially expressed genes between two m6A modified subtypes were defined as m6A modified subtype differential genes if they satisfied adjusted *p* < 0.05 and |logFC| > 0.5, and as m6A modified related genes if they satisfied adjusted *p* < 0.0001. Assessment of m6A modified subtype differential genes based on GO terms and KEGG pathway enrichment analysis (Q-value <0.05) (27). We used the “WGCNA” (Weighted Gene Co-expression Network Analysis) R package to identify the co-expression modules of m6A modified related genes (28). The dissimilarity of the module eigengenes was calculated to merge similar modules with a height cut-off value of 0.25, and a minimum module size set to 20 genes. The module eigengene (ME) is defined as the first principal component of a given module. Gene significance (GS) was denoted as the correlation of gene expression and the m6A modified subtypes. And the module membership (MM) was identified as the correlation between the gene expression and the ME. Pearson’s correlation was used to analyze the correlation. Genes with MM > 0.8 and GS > 0.6 were defined as module hub genes. The genes in the key modules were obtained, and the “MCC” algorithm was used to identify the top 20 central nodes for the protein–protein interaction (PPI) network, which was visualized using Cytoscape. The overlapping Genes of central nodes in PPI and hub genes in WGCNA were defined as m6A modified subtype marker genes.

The Nephroseq V5 tool^6^ was used to validate the correlation between m6A modified subtype marker genes and clinical indicators. In addition, NetworkAnalyst^7^, a database for network analysis, was utilized to predict transcription factors (TFs), miRNAs, and chemicals of clinically relevant marker genes, as well as to build biological interaction networks.

## Results

### Landscape of M6A regulators in DKD

**Figure 2:**
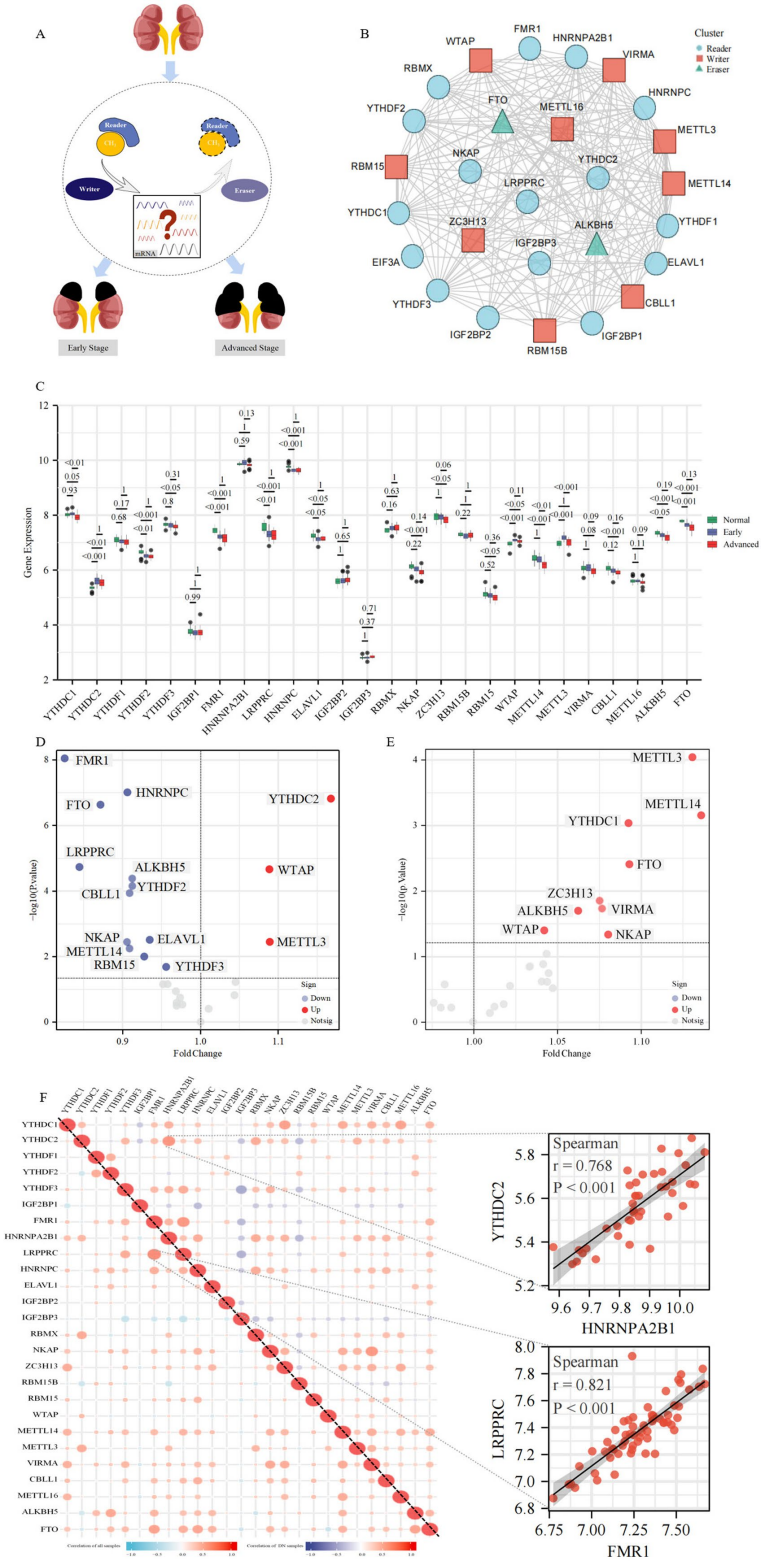
The landscape of m6A regulators in DKD. **(A)** Overview of the dynamic and reversible process of m6A modification, regulated by m6A “Writers,” “Readers” and “Erasers.” M6A modification is involved in the biological functions of DKD. **(B)** Protein–protein interaction (PPI) network composed of 27 m6A regulators. **(C)** Box plots show the expression levels of 26 m6A regulators in the glomeruli between the control group and different stages of DKD. Volcano plots show a summary of the differences in expression of 26 m6A regulators between glomerular samples from DKD vs. control patients **(D)** and the early vs. advanced DKD patients **(E)**, respectively. **(F)** Heatmaps show the correlation between the expression of 26 m6A regulators in all glomerular samples and DKD glomerular samples, respectively. The two scatter plots show the two pairs of m6A regulators with the highest correlation, respectively.

A total of 27 m6A regulators were included in this study, including 9 “Writers” (ZC3H13, RBM15B, RBM15, WTAP, METTL14, METTL3, VIRMA, CBLL1, METTL16), 16 “Readers” (YTHDC1, YTHDC2, YTHDF1, YTHDF2, YTHDF3, IGF2BP1, FMR1, HNRNPA2B1, LRPPRC, HNRNPC, ELAVL1, IGF2BP2, IGF2BP3, RBMX, NKAP, EIF3A) and 2 “Erasers” (ALKBH5, FTO). Figure 2A illustrates the inherent relationship between m6A-modified regulators and the occurrence and progression of DKD, which prompted to the purpose for our investigation. The regulatory interactions between these 27 m6A regulators are shown in Figure 2B.

To investigate the expression of m6A regulators in control and various stages of DKD, PBMC (Supplementary Figure 1A), whole kidney tissue (Supplementary Figure 1B), microdissected glomeruli (Figure 2C), and tubulointerstitial tissue (Supplementary Figure 1C) samples were evaluated, respectively. The data from the PBMC sample included in this study contained 28 cases of DKD and 10 healthy controls, in which patients with DKD were classified into early and advanced stages according to eGFR and urine protein levels, with a mean eGFR of 35 mL/min/1.73 m^2^ in the advanced stage. Data from whole kidney tissue samples contained 6 cases of early DKD, 21 cases of advanced DKD, and 9 paracancerous controls, in which patients with DKD were classified into early (mean eGFR 118 mL/min/1.73 m^2^) and advanced (mean eGFR approximately 64 mL/min/1.73 m^2^) according to eGFR and urinary albumin to creatinine ratio (UACR). In the glomerular microdissection sample data, there were 20 early DKD cases, 21 advanced DKD cases, and 20 paracancerous controls, in which patients with DKD were classified into early (mean eGFR 99 mL/min/1.73 m^2^) and advanced (mean eGFR 43 mL/min/1.73 m^2^) stages according to clinical and pathological features. The data in the renal tubular interstitial tissue sample contained 17 cases of DKD and 21 healthy controls in which DKD patients had eGFR <90 mL/min/1.73 m^2^. There were no significant differences in baseline data (e.g., age, BMI, HbA1c levels, etc.) between patients with early and advanced DKD in different tissue samples. Supplementary Figure 1A revealed that 8 m6A regulators were statistically different in PBMC samples against controls, and the majority of m6A regulators exhibited a dynamic tendency of up-regulation followed by down-regulation or vice versa as the disease progressed in DKD. The majority of m6A regulators exhibited significant differences across the three kinds of renal tissue samples (16/25 differential m6A regulators in whole kidney tissue, 16/26 differential m6A regulators in glomerular tissue, and 14/22 differential m6A regulators in tubulointerstitial tissue). Intriguingly, as the disease progressed, m6A regulators, specifically YTHDC2, YTHDF1, YTHDF2, IGF2BP2, and RBM15, in glomerular tissue showed a trend of changes inconsistent with whole kidney tissue, whereas tubulointerstitial tissue samples exhibited changes consistent with whole kidney tissue. This unexpected result likely happened because glomerular tissue in the whole kidney tissue sample was relatively limited. The majority of DKD-related lesions occur in the glomerulus, and the identification of m6A regulators was more comprehensive in the glomerular tissue samples included in this investigation, therefore the follow-up study focused mostly on glomerular tissue samples.

Notably, only YTHDC2, WTAP, and METTL3 expression was increased in DKD patients with m6A changed regulators in glomerular samples, whereas the most were downregulated compared to controls, with FMR1 expression level drop having the biggest and most statistically significant fold change (Figure 1D). Differential m6A regulators were all upregulated in expression in patients with early DKD compared to those with advanced DKD (Figure 1E). In the correlation study of 26 m6A regulators in glomerular tissue samples with and without controls, we observed a close link between the regulators, indicating that they have a coordinated effect (Figure 1F). FMR1 and LRPPRC were the most relevant m6A regulators in all glomerular samples, whereas HNRNPA2B1 and YTHDC2 were the most relevant in DKD glomerular samples.

### Immunological characteristics of DKD at various stages and the correlation with M6A regulators

**Figure 3:**
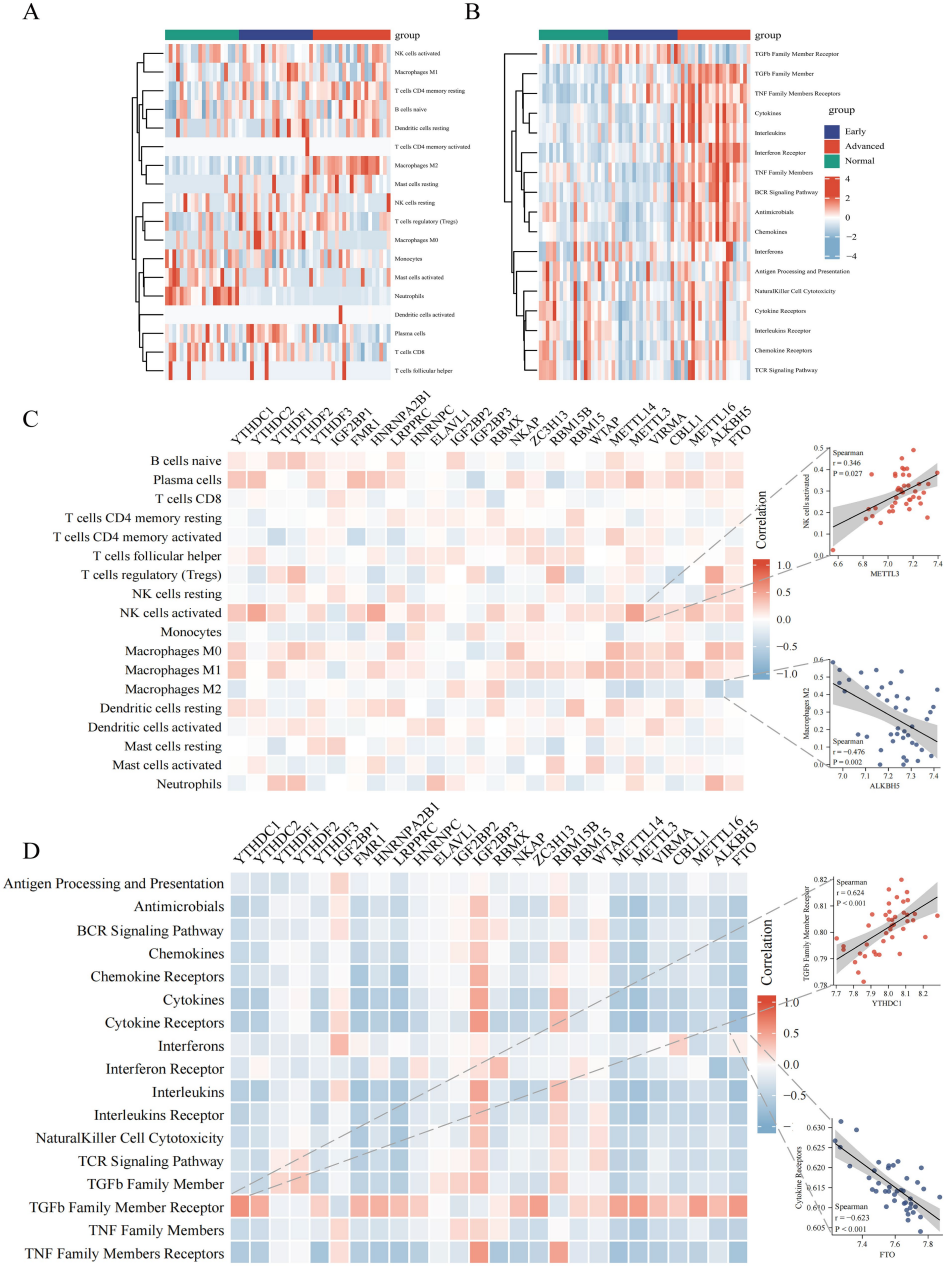
Correlation between m6A regulators expression and immune characteristics in DKD. Heatmaps of differential expression of infiltrating immune cells abundance **(A)** and immune response gene sets activities **(B)** in glomerular samples (removal of B cells memory, T cells CD4 naive, T cells gamma delta and Eosinophils, which were not expressed in all samples). **(C)** Heatmap of the correlation between 26 m6A regulators and 18 immunocytes. The two respective scatterplots show the m6A modified regulator and immunocyte with the highest positive or negative correlation. **(D)** Heatmap of the correlation between 26 m6A regulators and 17 immune response gene sets. The two respective scatterplots show m6A regulators and immune response gene sets with the highest positive or negative correlation.

To investigate the changing immune characteristics of DKD, the CIBERSORT algorithm was utilized to compare the expression of infiltrating immune cells abundance in glomerular samples from healthy controls and DKD patients at various stages (Supplementary Figure 2A). Memory B cells, naive CD4+ T cells, γδ T cells, and eosinophils were excluded from the expression differential analysis due to their lack of expression in all samples. The differential analysis revealed significant shifts in macrophages among the intrinsic immune response cells, with an increase in macrophage M0 and M1 in the early DKD stage and in macrophage M2 in the advanced DKD stage. Activated mast cells and neutrophils were significantly reduced in DKD. Regulatory T cells were elevated in early DKD, but plasma cells and CD8+ T cells were decreased in advanced DKD (Figure 3A and Supplementary Figure 2A). The immune response gene sets dominated by cytokines, interleukins, chemokines, TGFβ family members, TNF family members, and BCR signaling pathway were revealed to be considerably active in advanced DKD. Chemokine receptors and cytokine receptors revealed a transitory decline in early DKD, but TNF family member receptors and interferon receptors were increasingly active as DKD progressed.

To study further the relationship between m6A regulators and immune characteristics, we evaluated their association. Both immune cell infiltration and immune response gene sets were related with m6A regulators, according to the Heatmaps. Activated NK cells had the most positive correlation with METTL3, whereas macrophage M2 had the strongest negative correlation with ALKBH5 (Figure 3C). TGFβ family member receptor had the strongest positive association with YTHDC1, cytokine receptors had the strongest negative association with FTO, and the majority of immune response gene sets had negative associations with m6A regulators (Figure 3D).

### Potential of M6A regulators model to identify early DKD

**Figure 4:**
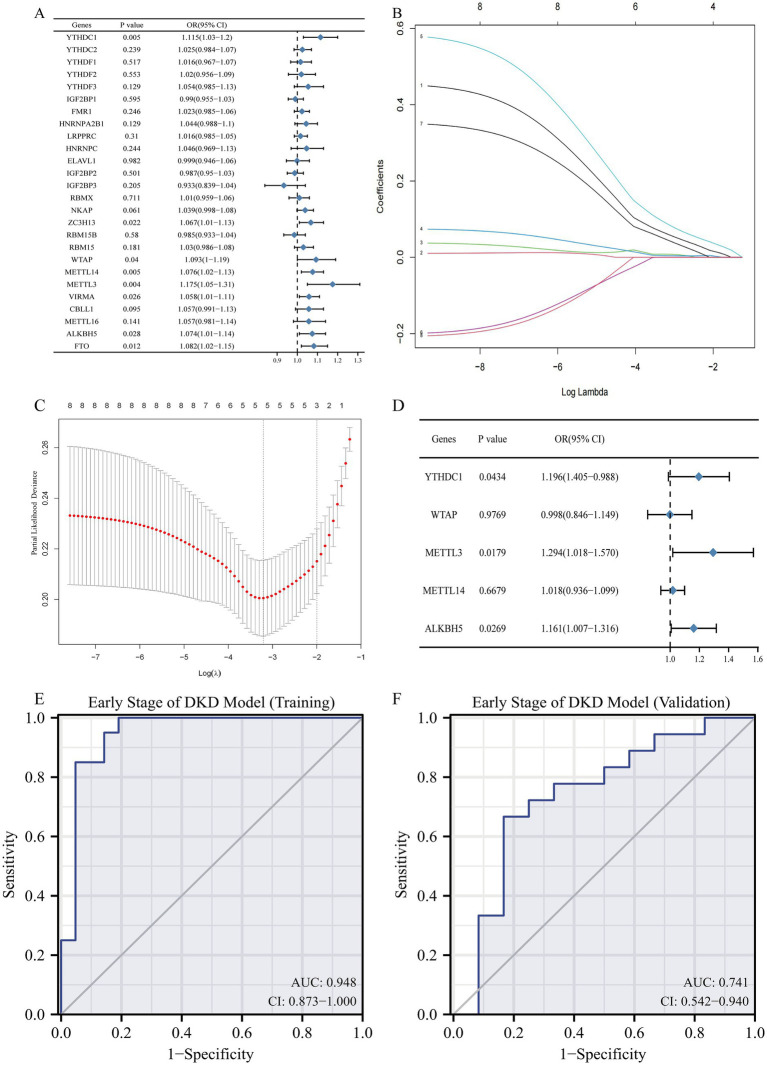
The m6A regulators have the potential to identify the early and advanced DKD. **(A)** Univariate logistic regression revealed that eight m6A regulators were independently related with early DKD (*p* < 0.05). **(B,C)** Feature selection by LASSO regression model. **(B)** Least absolute shrinkage and selection operator (LASSO) coefficient distributions for 8 early DKD-associated m6A regulators. **(C)** 10-fold cross-validation was conducted to select the best model in the LASSO regression. The partial likelihood deviance is plotted against log (*λ*), where λ is the tuning parameter. The dotted vertical lines are drawn at the optimal values by minimum criteria and 1-SE criteria. Five features with non-zero coefficients were selected by optimal lambda. **(D)** Multivariate logistic analysis distinguished three independent factors to model the identification of early DKD. **(E)** The ability of the m6A regulators model to discriminate the early DKD was analyzed using ROC curves and evaluated with AUC values. Model validation was also performed with GSE142152 dataset **(F)**.

To research the role of m6A regulators in the progression of DKD, we developed a model of m6A regulators. Eight modified m6A regulators were identified to be related with early DKD by the use of univariate logistic regression, which were YTHDC1, ZC3H13, WTAP, METTL14, METTL3, VIRMA, ALKBH5, and FTO (Figure 4A). Next, LASSO regression was utilized to further filter eight early DKD-associated m6A regulators, obtaining five m6A regulators with non-zero coefficients (Figures 4B,C). Lastly, putting the LASSO regression results into multi-factor logistic regression showed that YTHDC1, METTL3, and ALKBH5 were independent correlates of early DKD, and the m6A regulators model was developed for further research (Figure 4D). The AUC of the training set (GSE96804) for this m6A regulators model was 0.948, indicating that the model identified between early and advanced DKD efficiently (Figure 4E). Considering that whole kidney tissue samples were not tested for YTHDC1 regulators and there were no DKD clinical staging data in tubulointerstitial tissue, PBMC sample data (GSE142153) were used as an independent external validation set to evaluate the extrapolation of the model. Ultimately, the AUC of the validation set was 0.741 (Figure 4F), indicating that the m6A regulators model is promising as a classifier of early DKD and advanced DKD and deserves to be further studied. Unfortunately, our study was unable to develop a diagnostic model of m6A regulators adequate for identifying DKD from controls.

### Identification of M6A modified subtypes

**Figure 5:**
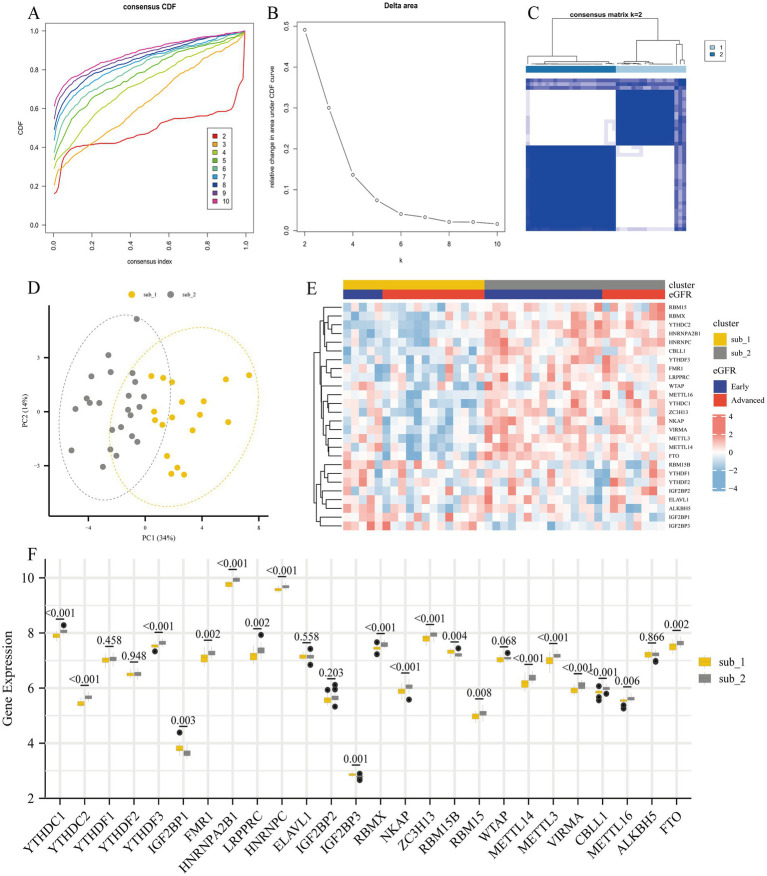
Unsupervised consensus clustering analysis of 26 m6A regulators for identification of m6A modified subtypes. **(A)** Consensus clustering of cumulative distribution function (CDF) for *k* = 2–10. **(B)** Elbow plot shows relative change in area under CDF curve. **(C)** Consensus clustering matrix for *k* = 2. **(D)** Principal component analysis (PCA) of two m6A subtypes in DKD. **(E)** Heatmap showing the distribution of different DKD stages and m6A gene profiles in the two m6A modified subtypes. **(F)** The 26 m6A regulators showed differences in the two m6A modified subtypes.

To investigate the regulatory mechanisms of m6A regulators during the progression of DKD, we performed an unsupervised consensus clustering analysis of DKD glomerular samples based on the expression of 26 m6A regulators (Figures 5A–C). A total of 2 different m6A modified subtypes were identified, with 18 DKD cases in subtype 1 and 23 DKD patients in subtype 2. The two m6A modified subtypes were significantly different in PCA (Figure 5D). In the heatmap, substantial variations in the expression profiles of m6A regulators were seen between the two subtypes, with early DKD being primarily dispersed in subtype 2 (Figure 5E). YTHDC1, YTHDC2, YTHDF3, FMR1, HNRNPA2B1, LRPPRC, HNRNPC, NKAP, ZC3H13, RBM15, METTL14, METTL3, VIRMA, CBLL1, METTL16, and FTO were highly expressed in subtype 2, whereas IGF2BP1, IGF2BP3, and RBM15B were highly expressed in subtype 1 (Figure 5F).

### Immune characteristics and biological functions of M6A modified subtypes

**Figure 6:**
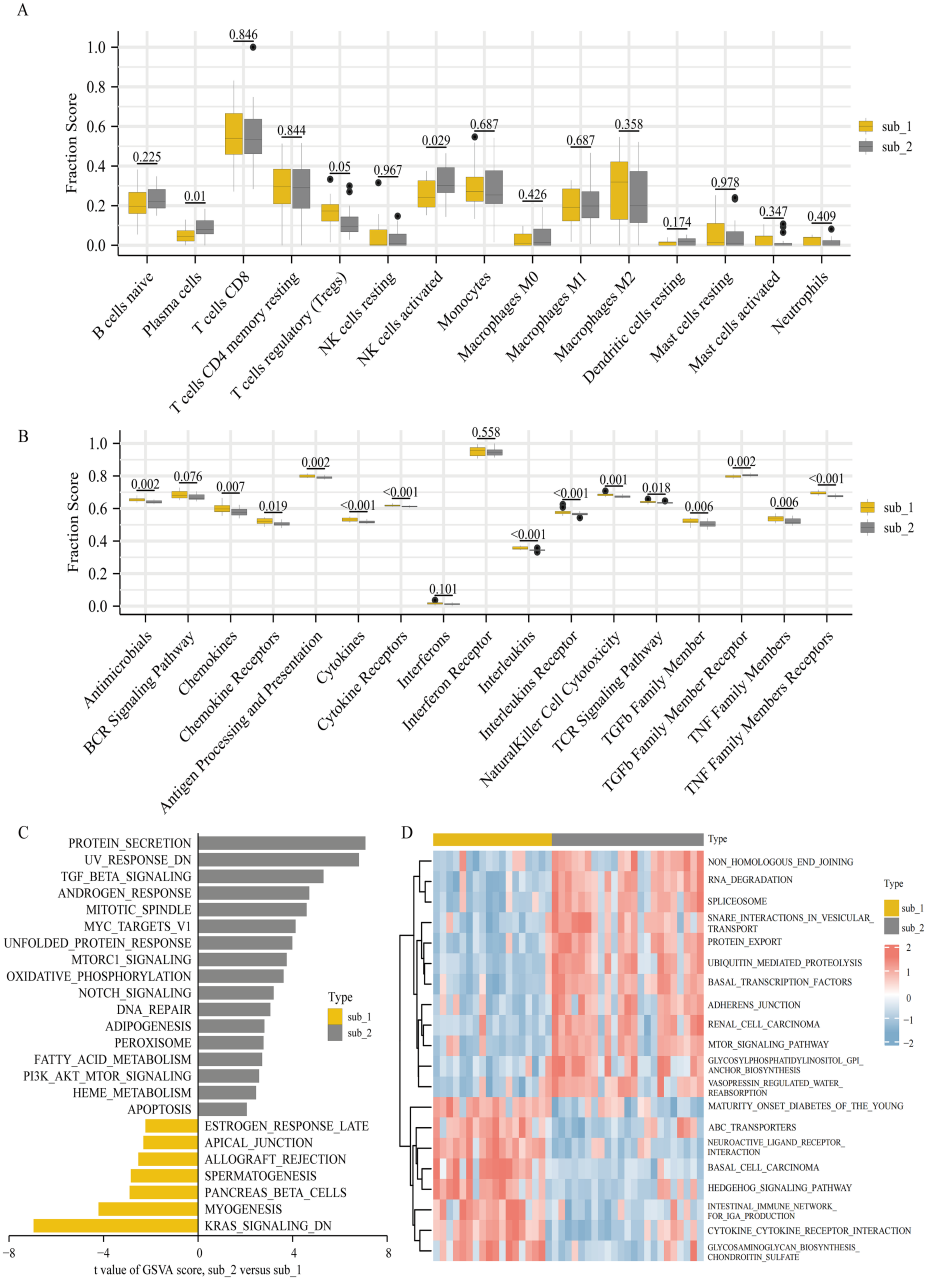
Diversity of immune characteristics and biological function enrichment analysis in two m6A modified subtypes. **(A)** Differences in the abundance of infiltrating immunocytes in the two m6A modified subtypes (removal of T cells CD4 memory activated, T cells follicular helper and dendritic cells activated where low expression does not allow comparison of differences). **(B)** Differences in the activities of 17 immune response gene sets in two m6A modified subtypes. The differences of HALLMARKS pathway **(C)** and KEGG pathway **(D)** enrichment scores between m6A modified subtypes.

To explore the differences in immune characteristics between the two m6A modified subtypes, we evaluated the abundance of immune infiltrating cells and the scoring of immune response gene sets. It was shown that plasma cells and activated NK cells were much more prevalent in subtype 2, whereas Tregs were comparatively more prevalent in subtype 1. However, there was minimal variation in the amount of immune infiltrating cells between the two subtypes (Figure 6A). The 2 subtypes showed significant differences in the scoring of the immune response gene sets. Except for the TGF*β* family member receptor, which was more active in subtype 2, the rest of the differentially immune response gene sets were less active in subtype 2 (Figure 6B).

We performed a GSVA to further research the differences in biological functional pathways between the 2 subtypes. Enrichment in the HALLMARKS pathway revealed that protein secretion, genes with reduced UV sensitivity, and TGFβ signaling pathway were more enriched in subtype 2, whereas myogenesis, down-regulated KRAS signaling pathway and pancreatic β cells were more enriched in subtype 1 (Figure 6C). Significant differences in KEGG pathway enrichment were observed between the two subtypes, with cytokine-cytokine receptor interaction, intestinal immune network for IgA production, hedgehog signaling pathway, and glycosaminoglycan biosynthesis chondroitin sulfate enriching predominantly in subtype 1 and RNA degradation, protein export, ubiquitin mediated proteolysis, and mTOR signaling pathway enriching mainly in subtype 2 (Figure 6D).

### Identification and clinical relevance of M6A modified subtypes marker genes

**Figure 7:**
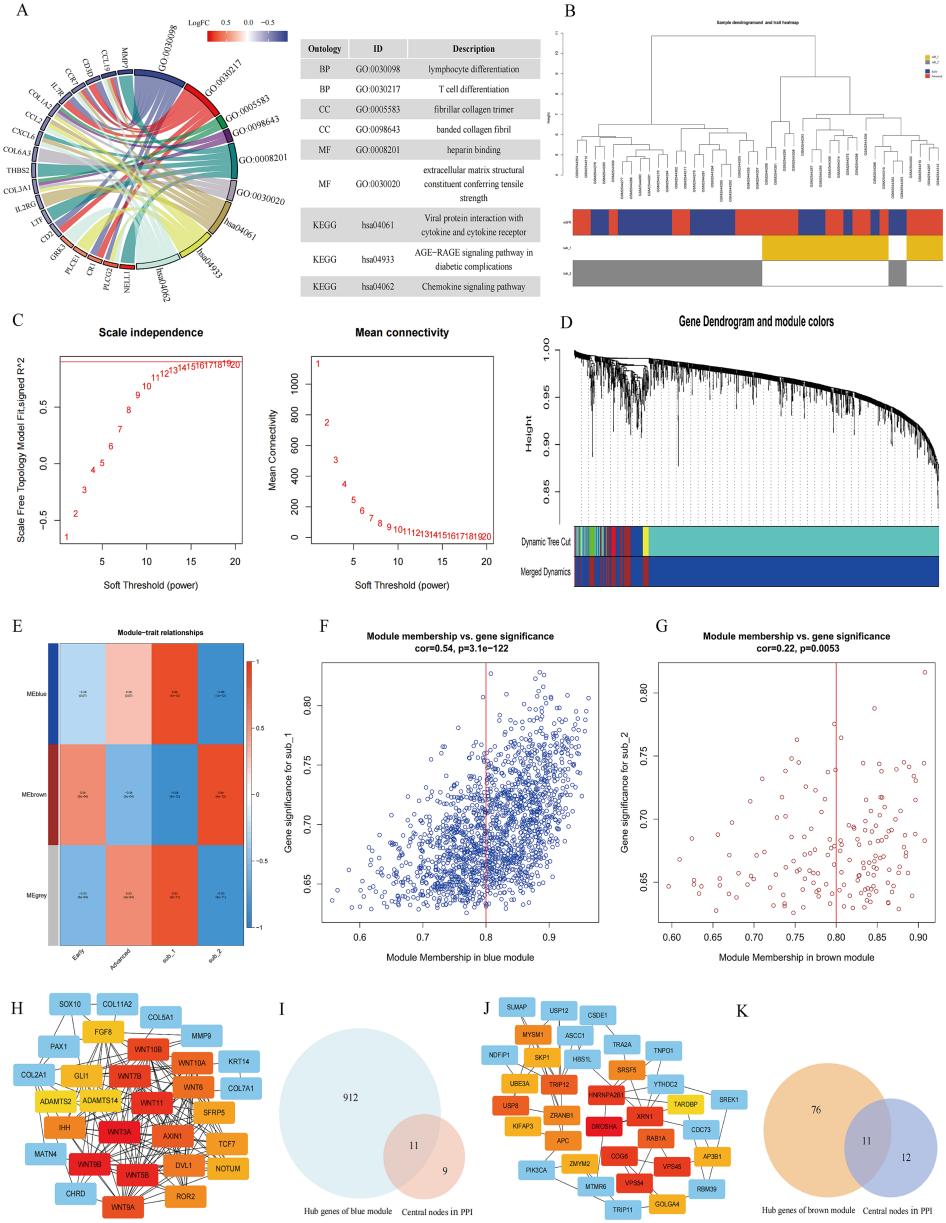
Identification and functional analysis of modified subtypes marker genes. **(A)** GO and KEGG enrichment analysis of modified subtype differential genes. **(B)** Cluster dendrogram of the two m6A modified subtypes and clinical stages of DKD. **(C)** Scale-free fitting index analysis and mean connectivity of soft threshold power from 1 to 20. **(D)** Clustering dendrogram of m6A modified related genes. Based on the dynamic tree cut, the genes are clustered into different modules by hierarchical clustering, and then the modules with similarity greater than 0.75 are merged to reduce the complexity of the network. Each color represents one module, and finally 3 modules are identified. **(E)** Correlation heatmap between module eigengenes and m6A subtypes and clinical features. Scatterplots of gene significance (GS) for m6A subtype 1 versus module membership (MM) in the blue module **(F)** and GS for m6A subtype 2 versus MM in the brown module **(G)**. The MM of these genes >0.8 and their GS > 0.6 for the points means that these points are the hub genes of the module. PPI network analysis of blue module **(H)** and brown module **(J)** genes, visualized with Cytoscape, where the central nodes in the PPI are marked in red, orange and yellow. Venn diagram of m6A subtype 1 (blue module) **(I)** marker genes and m6A subtype 2 (brown module) **(K)** marker genes. The central nodes of PPI overlap with their corresponding hub genes in the blue **(I)** and brown modules **(K)**, respectively.

To further understand the biological processes of genes affected by m6A regulators, we identified m6A modified subtype differential genes and performed GO/KEGG enrichment analysis on these genes. A total of 73 m6A modified subtype differential genes with GO functional enrichment were mainly focused on immune cell differentiation, collagen component formation, heparin binding and enhanced extracellular matrix resistance. The top 3 categories enriched in KEGG pathways were viral protein interaction with cytokine and cytokine receptor, AGE-RAGE signaling pathway in diabetic complications, and chemokine signaling pathway (Figure 7A).

To further explore the co-expression relationships between genes, 1768 m6A modified related genes were included in the WGCNA to identify modular hub genes (Figures 7B–D). A total of three modules were identified as blue, brown and meaningless gray modules (Figure 7E). The blue module had the highest positive correlation (*R*^2^ = 0.86) with m6A modified subtype 1, meanwhile the brown module had the highest positive correlation (*R*^2^ = 0.84) with m6A modified subtype 2. Interestingly, the early DKD was also positively correlated with the brown module. A total of 923 hub genes in the blue module and 87 hub genes in the brown module were found (Figures 7F,G). Finally, the top 20 central nodes of the PPI of the blue and brown module genes were overlapped with the hub genes of the respective modules, and 11 marker genes for each of the two subtypes were discovered (Figures 7H–K). The subtype 1 marker genes were WNT11, WNT7B, WNT10B, AXIN1, WNT10A, DVL1, ROR2, SFRP5, NOTUM, FGF8, and ADAMTS14. And VPS54, XRN1, TRIP12, ZRANB1, APC, ZMYM2, GOLGA4, KIFAP3, SKP1, AP3B1, and PIK3CA were the subtype 2 marker genes.

**Figure 8:**
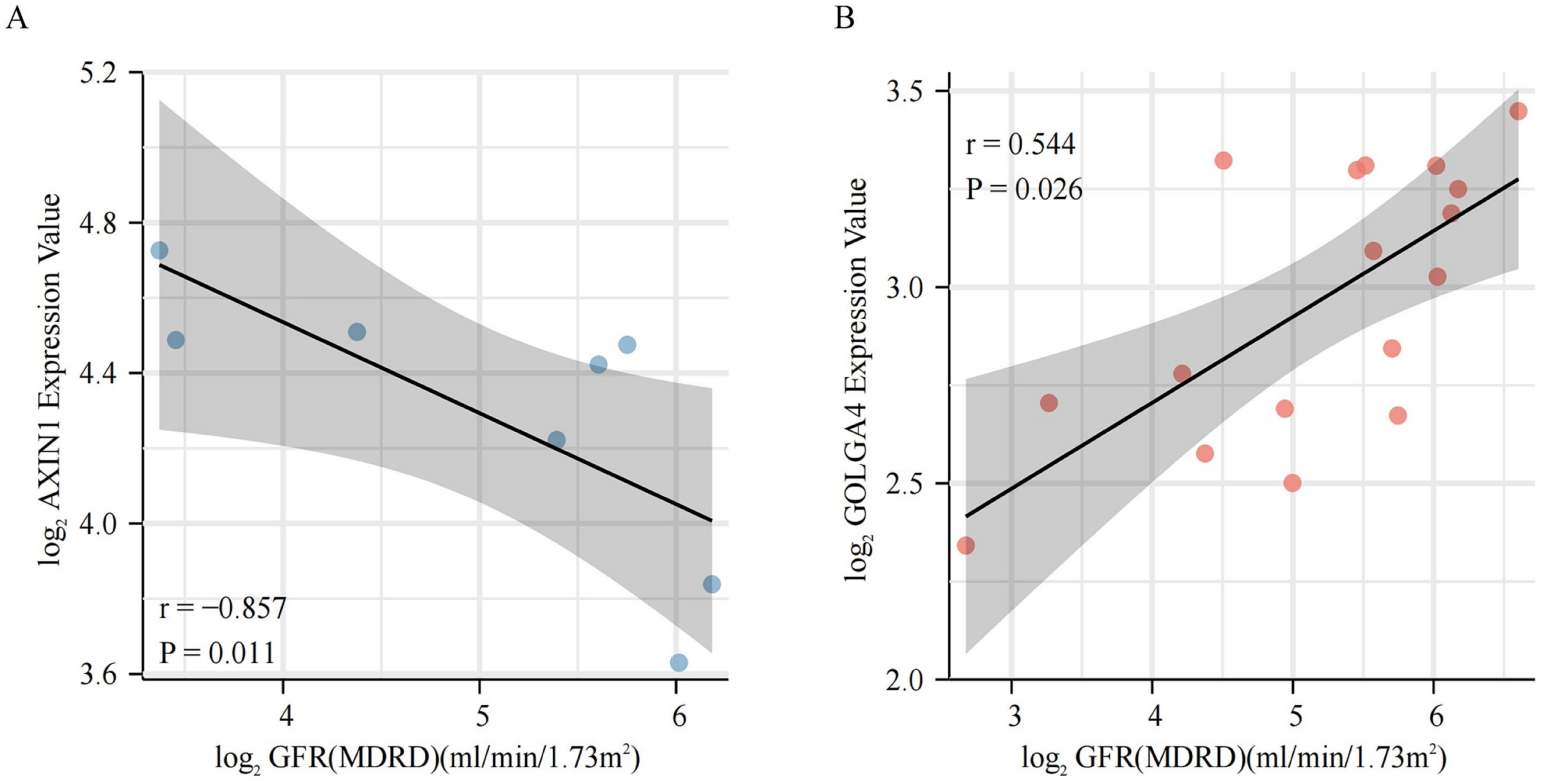
Clinical relevance of m6A modified subtypes marker genes. **(A,B)** Relationship between m6A modified subtypes marker genes and glomerular filtration rate.

To further elucidate the association of these m6A modified subtype marker genes with DKD disease, we correlated the marker genes with clinical data from the Nephroseq database. It was found that the AXIN1 gene was negatively connected with GFR in subtype 1, whereas the GOLGA4 gene was positively correlated with GFR in subtype 2 (Figures 8A,B). The regulatory network maps of transcription factors, miRNAs, and chemicals for the two m6A modified subtypes marker genes, which may be exploited for future research, are displayed in Supplementary Figure 3.

## Discussion

Diabetic kidney disease is a prevalent microvascular complication of diabetes mellitus that is characterized by rapid progression and a poor prognosis, causing a serious risk to human health and life. Different therapeutic options are available for various stages of DKD (29), and the understanding of its pathogenesis is still limited, thus it is necessary to identify alternative biomarkers and possible molecular targets for the prevention and treatment of DKD.

From the perspective of m6A modifications, this article demonstrates the progression mechanism of DKD. First, when compared with the control group, we observed significant differences in the expression of most m6A regulators in DKD, whether PBMC or all or part of kidney tissues. Throughout the progression of DKD, the stages of change in m6A regulators are inconsistent. Some regulators, such as YTHDC2, YTHDF2, and FMR1 showed significant changes in the early stage of DKD, some regulators, such as YTHDC1 and METTL14 exhibited significant changes in the advanced stage of DKD, and some regulators, such as METTL3 demonstrated dynamic changes, which suggested that m6A regulators may play a role in the development of the DKD process. DKD is a metabolic disease involving inflammatory immunology, and previous study have demonstrated an increase in inflammatory cells in the kidneys of DKD patients, with leukocyte counts that are seven to eight times higher than in healthy kidneys (30). Macrophage infiltration is one of the distinctive characteristics of DKD and is notably increased in the glomerular tissue of the majority of DKD patients (31). In this study, macrophage infiltration was also observed in the evaluation of immune characteristics, and the type of infiltrating macrophages differed in different stages. In the early stage, M1 macrophage infiltration predominated, whereas M2 macrophage infiltration dominated in the advanced stage. According to previous research, M1 macrophages can damage the kidneys of DKD patients by producing pro-inflammatory cytokines (e.g., IL-1β and IL-23), chemokines, and reactive oxygen species, whereas M2 macrophages generally function as anti-inflammatory cells and are involved in immunosuppression, tissue repair, and tumor progression (32, 33). The M1-to-M2 transition of macrophages has been found in both the AKI mouse model and the renal fibrosis UUO model, as well as the presence of CD 163+ M2 macrophages has been shown to accelerate fibrosis and disease progression in DKD (34–37). Mast cells have been involved in interstitial kidney injury in people with DKD, which is associated with renal fibrosis (38). In this work, however, both innate immune response cells (activated mast cells and neutrophils) and CD8+ T cells involved in particular immunological response were observed to be decreased in DKD, but the detailed mechanism remains uncertain. Various diseases stages elicit different immune responses. In this research, immune response gene sets containing cytokines, chemokines, interleukins, TGFβ family members, and TNF family members were active in advanced DKD, while cytokine receptor and interleukin receptor gene sets were changed in early DKD.

Then, what causes the dynamic changes in immune characteristics with the progression of DKD? The subsequent correlation heatmap findings provide the possible explanation. m6A regulators were significantly associated with a range of immune cells and immune responses, as indicated by correlation heatmaps. The majority of regulators are associated with activated NK cells, M1 and M2 macrophages, cytokines, and TGF family member receptors, while METTL3, METTL14, FTO, and IGF2BP3 regulate multiple immune cells and immune responses. Growing evidence confirms that m6A regulators play an important role in the immune response (14, 39–41). In the glomerulus, m6A regulators may contribute to DKD development by regulating diverse immune cells and immunological responses. The correlation between m6A regulators and diverse immune cells was found to be weaker than the immune response in this investigation. This finding is similar to that of previous studies, and the author attributes the result to a technical limitation (42).

Recent research on urinary m6A shown that m6A levels in the urine of DKD patients gradually decreased as the disease progressed, and the author concluded that urinary m6A levels had the potential to serve as a biomarker for early identification and monitoring of DKD (43). Similarly, in this study, a model of m6A regulators including YTHDC1, METTL3 and ALKBH5 was developed by logistic-LASSO regression to identify early DKD. In glomerular tissue, the model was able to identify early and advanced DKD well, and when verified using PBMC samples, the same good identification ability was observed. The results suggest that m6A regulators have the potential to be used as biomarkers for early DKD diagnosis. However, this study was unable to develop a model to identify between DKD and non-DKD. It revealed the presence of dynamic changes in m6A regulators at various stages of DKD and indicated that m6A regulators may play a key role in regulating disease development. Despite the fact that the diagnostic efficacy of this model was lower in PBMC than in glomerular samples (probably due to tissue type heterogeneity), it still suggests that m6A methylation modifications play a significant regulatory function in the progression of DKD.

To further investigate the regulation of m6A methylation modification during the progression of DKD, unsupervised consensus clustering analysis was performed on DKD samples based on the expression of m6A regulators. A total of two m6A modified subtypes were identified, whose expression of m6A regulators differed significantly. In subtype 2, the majority of m6A regulators (including “Writer,” “Eraser,” and “Reader”) were up-regulated, showing a hypermetabolic state of methylation and demethylation. We found a larger number of subtype 2 individuals with early DKD, indicating the fact that the model of m6A modified subtypes is different from clinical categorization but associated with the degree of disease progression.

In terms of immune cells, the majority of infiltrating immune cells did not differ significantly between the two subtypes, whereas in terms of immune responses, the majority of immune responses demonstrated decreased activity in subtype 2, consistent with the trend observed for immune responses in early DKD. M6A modified subtype 2 was considerably enriched in protein secretion, TGFβ signaling pathway, PI3K/AKT/mTOR signaling pathway, Notch signaling pathway, oxidative phosphorylation, fatty acid metabolism and apoptosis, while subtype 1 was significantly enriched in myogenesis. GO/KEGG enrichment study indicated variations in cell differentiation, collagen fiber composition, and chemokine pathways between the two subtypes. Despite the fact that most immune response activities of subtype 2 are less active than those of subtype 1, subtype 2 is enriched in signaling pathways such as inflammation, proliferation, and apoptosis, whereas subtype 1 is enriched in myogenesis, collagen formation, and fibrosis. The mechanisms of the two subtypes have different foci, and the classification based on the m6A regulators model could be considered as an alternative classification for DKD, as well as clinically targeting therapy according to different molecular mechanisms in different subtypes and guiding different stages of medication to improve efficacy.

In this study, m6A modified subtypes marker genes were obtained by WGCNA and modular PPI analysis. It can be found that WNT11, WNT7B, WNT10B, AXIN1, WNT10A, DVL1, ROR2, SFRP5 and NOTUM marker genes in subtype 1 are mainly involved in the Wnt signaling pathway. According to whether they rely on *β*-catenin or not, Wnt signaling pathway is divided into typical signaling pathway (β-catenin-dependent) and atypical signaling pathway (β-catenin-independent) (44, 45). It has also been revealed that the Wnt signaling pathway is activated during renal injury and is involved in regulating renalintrinsic cells injury and renal fibrosis, and it is now considered as a crucial regulator in the development of DKD (46–48). FGF8 is a subtype of the FGF family of fibroblast growth factors that binds to FGFR and participates in paracrine secretion-mediated biological activity (49). It has been reported that members of the FGF family (FGF1, FGF2, FGF21, FGF23, etc.) are involved in metabolic processes in DKD, but no research relating FGF8 subtype to DKD have been published (50–52). ADAMTS14 is a subtype member of the ADAMTS (a disintegrin-like and metalloprotease domain with thrombospondin type 1 repeats) metalloproteinase family, which forms procollagen N-protease with ADAMTS2 and ADAMTS3 to degrade type I, II, III, and V procollagen and promote collagen fibers formation, and participate in coagulation processes, growth and evolution, signal transduction, and tumor progression (53, 54).

In subtype 2, the adenomatous polyposis coli (APC) gene negatively regulates the WNT signaling pathway by promoting phosphorylation, ubiquitination and protein degradation of *β*-catenin. It has been reported that upregulation of APC rescues the effects of miR-499-5p overexpression on kidney injury in mice with DKD (55). The PIK3CA gene produces a protein that is the catalytic subunit of phosphatidylinositol-3-kinase (PI3K), and the PI3K-AKT–mTOR pathway regulated by PI3K has been widely recognized to have a role in kidney injury and DKD progression (56). The remaining marker genes were not reported to be directly associated with DKD. VPS54 participates in the transport and sorting of several proteins inside cells. XRN1 is involved in RNA degradation. The protein encoded by the TRIP12 is an E3 ubiquitin-protein ligase that plays a role in the DNA damage response. ZRANB1 is involved in fat metabolism regulation. The zinc finger protein encoded by the ZMYM2 gene may serve as a transcription factor. It is thought that KIFAP3 serves as a linker between human chromosome-associated polypeptide (HCAP) and KIF3A/B, a kinesin superfamily protein in the nucleus, and that it plays a role in the interaction of chromosomes with an ATPase motor protein. Skp1 (S-phase kinase-associated protein 1-*Homo sapiens*) is an adapter protein of the SCF (Skp1-Cullin1-Fbox) complex, which is involved in cell cycle regulation. The protein encoded by AP3B1 is part of the heterotetrameric AP-3 protein complex which interacts with the scaffolding protein clathrin. GOLGA4 is a Golgi matrix protein involved in glycosylation and transport of proteins and lipids.

Nephroseq database clinical data further verified the direct clinical significance of marker genes in m6A modified subtypes. In subtype 1, the AXIN1 gene was shown to be negatively correlated with the GFR, and in subtype 2, the GOLGA4 gene was found to be positively associated with the GFR. Decreasing AXIN1 or increasing GOLGA4 levels may ameliorate DKD, and gene-TF-miRNA and gene-compound regulatory networks are directions for further research. Although just these two genes were shown to be significantly related to GFR in the Nephroseq database, other marker genes are still worth investigating. Previous studies by our team shown that METTLL14 causes *α*-klotho methylation, resulting in kidney injury (16). While klotho can directly and competitively bind Wnt ligands to inhibit the activation of this pathway, which is equivalent to m6A regulators interact on the Wnt signaling pathway indirectly by mediating α-klotho methylation (57, 58). Thimoteus Speer noted that it is interesting to determine the exact timing of the possible beneficial effects of KP6 (klotho-derived peptide 6) during renal injury (59, 60). Our study provides answers to the aforementioned concerns and suggests that KP6 can be used in subtype 1 to enhance effectiveness, hence reducing kidney injury.

## Conclusion

This is the first study to analyze the association between m6A modification and immune characteristics in DKD. In addition to the phenotypic level, the m6A regulators model describes the progression of DKD at the molecular level. Certainly, there are some limitations to this study. First, the sample size was insufficient, and neither the clinicopathological data nor the categories of m6A regulators were comprehensive. In this study, the glomerular dataset includes a total of 41 DKD patients and 20 controls, however, more cases are required for further study. In the collection of total kidney tissue samples and glomerular samples, the variety of m6A-modified regulators is rather limited. Due to a lack of clinicopathological data, this aspect of m6A modification cannot be thoroughly studied. Second, no direct experimental validation has been conducted. Through bioinformatics analysis, our study identified marker genes for different m6A modified subtypes, nevertheless, animal and cell research are still required to confirm the exact molecular mechanism. Furthermore, the marker genes in our study were validated by Nephroseq database data, which increased the credibility of the results.

In conclusion, our study developed a model for early DKD identification. The model of m6A modified subtype have the ability to categorize DKD at the molecular level and are anticipated to perform as an alternate classification approach. AXIN1 and GOLGA4 are potential biomarkers for targeted therapy. We comprehensively evaluated the potential regulatory mechanisms of m6A modification in the progression of DKD, providing new insights into DKD and inspiring more effective therapy methods.

## Data Availability

The original contributions presented in the study are publicly available. This data can be found here: https://www.ncbi.nlm.nih.gov/geo/ accession numbers: GSE142153, GPL6480, GSE142025, GPL20301, GSE96804, GPL17586, GSE104954, GPL22945, GPL24120.

## Footnotes

^1^ https://www.ncbi.nlm.nih.gov/geo/

^2^ https://string-db.org/

^3^ https://cytoscape.org/

^4^ https://www.immport.org/shared/home

^5^ https://www.gsea-msigdb.org/gsea/msigdb

^6^ http://v5.nephroseq.org/

^7^ http://www.networkanalyst.ca
