# Supplementary figures and images for: Diabetic kidney disease: m6A modification as a marker of disease progression and subtype classification
Source: Front Med (Lausanne). 2025 Mar 4;12:1494162. doi: 10.3389/fmed.2025.1494162 (PMC11914134; doi:10.3389/fmed.2025.1494162)

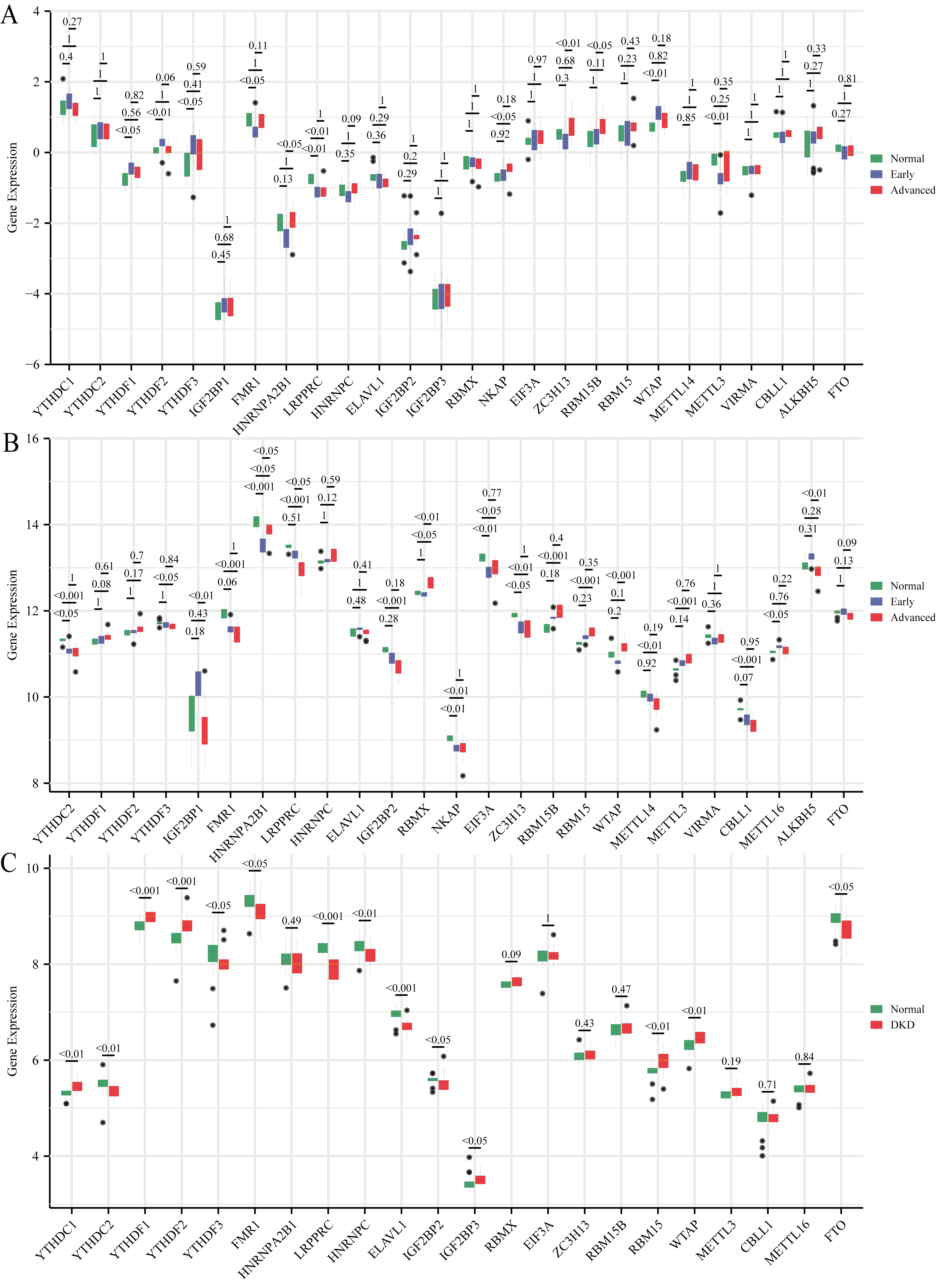

Supplement: Supplementary file 1 [file Image_1.TIF]

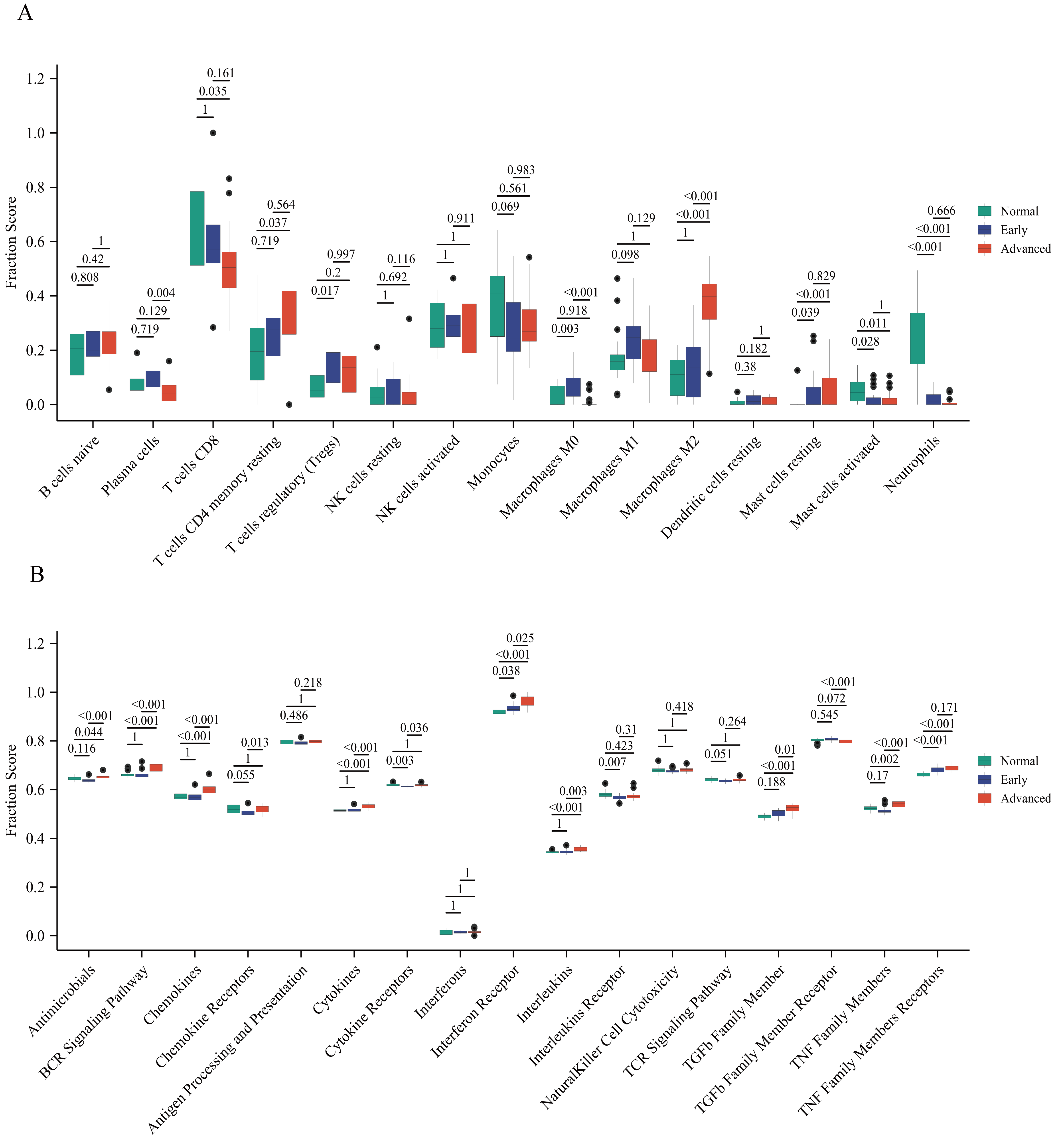

Supplement: Supplementary file 2 [file Image_2.TIF]

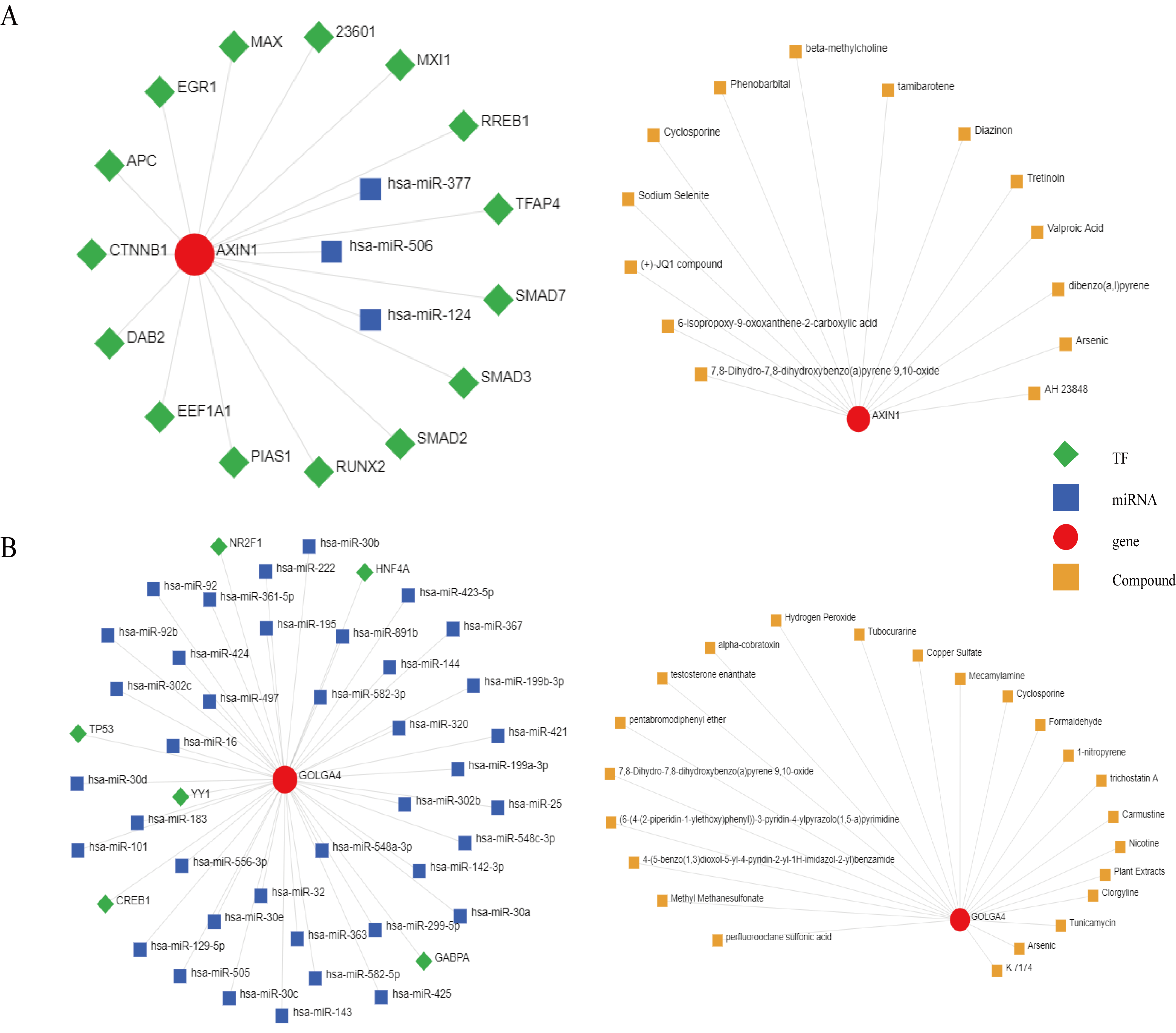

Supplement: Supplementary file 3 [file Image_3.TIF]
